# Interactive Local Super-Resolution Reconstruction of Whole-Body MRI Mouse Data: A Pilot Study with Applications to Bone and Kidney Metastases

**DOI:** 10.1371/journal.pone.0108730

**Published:** 2014-09-29

**Authors:** Oleh Dzyubachyk, Artem Khmelinskii, Esben Plenge, Peter Kok, Thomas J. A. Snoeks, Dirk H. J. Poot, Clemens W. G. M. Löwik, Charl P. Botha, Wiro J. Niessen, Louise van der Weerd, Erik Meijering, Boudewijn P. F. Lelieveldt

**Affiliations:** 1 Department of Radiology, Leiden University Medical Center, Leiden, the Netherlands; 2 Percuros B.V., Enschede, the Netherlands; 3 Departments of Radiology and Medical Informatics, Erasmus MC — University Medical Center Rotterdam, Rotterdam, the Netherlands; 4 Department of Intelligent Systems, Delft University of Technology, Delft, the Netherlands; 5 Quantitative Imaging Group, Faculty of Applied Sciences, Delft University of Technology, Delft, the Netherlands; 6 Department of Human Genetics, Leiden University Medical Center, Leiden, the Netherlands; Northwestern University Feinberg School of Medicine, United States of America

## Abstract

In small animal imaging studies, when the locations of the micro-structures of interest are unknown *a priori*, there is a simultaneous need for full-body coverage and high resolution. In MRI, additional requirements to image contrast and acquisition time will often make it impossible to acquire such images directly. Recently, a resolution enhancing post-processing technique called super-resolution reconstruction (SRR) has been demonstrated to improve visualization and localization of micro-structures in small animal MRI by combining multiple low-resolution acquisitions. However, when the field-of-view is large relative to the desired voxel size, solving the SRR problem becomes very expensive, in terms of both memory requirements and computation time. In this paper we introduce a novel *local* approach to SRR that aims to overcome the computational problems and allow researchers to efficiently explore both global and local characteristics in whole-body small animal MRI. The method integrates state-of-the-art image processing techniques from the areas of articulated atlas-based segmentation, planar reformation, and SRR. A proof-of-concept is provided with two case studies involving CT, BLI, and MRI data of bone and kidney tumors in a mouse model. We show that local SRR-MRI is a computationally efficient complementary imaging modality for the precise characterization of tumor metastases, and that the method provides a feasible high-resolution alternative to conventional MRI.

## Introduction

In pre-clinical small animal research on complications of cancer, imaging modalities like bioluminescence (BLI), CT, and MRI are conventionally used. Such imaging techniques allow non-invasive studies on the metastatic behavior of tumors [1]. BLI gives an indication of metastatic tumor growth anywhere in the body (*e.g.* bones, liver, and lungs), but the spatial resolution is not sufficient to distinguish between lesions located in close proximity to each other and to actually localize all individual metastatic processes in an organ. CT gives excellent contrast in calcified tissue and can be used to study tumor-induced changes in the bone, but it is less suitable to image organs such as liver and lungs due to lack of soft tissue contrast. MRI is the preferred imaging modality for imaging liver and lung metastases as it gives sufficient anatomical detail and good contrast between the organs and tumor masses. So, whereas BLI can be used to indicate the total tumor burden in an organ, MRI provides information on the location, size, and number of metastatic lesions in the organ. Since the location of the tumors is not known *a priori*, whole-body imaging is used, and CT, MRI, and BLI together aim to provide a comprehensive picture of the tumor and metastases development and spread in the entire body.

The sensitivity of MRI for small lesions is, however, relatively low compared to BLI, and the most robust pre-clinical protocols are still 2D MRI experiments with relatively thick slices. This slice thickness results in a large partial volume effect, making precise detection and localization of tumors difficult, especially for early stage tumors and micro tumors [2]. Recently, a resolution enhancing post-processing technique called super-resolution reconstruction (SRR) has been demonstrated to improve visualization and localization of micro-structures in molecular MRI [3], [4]. SRR computes a high-resolution image by combining a number of low-resolution images with varying fields-of-view (FOV). In a metastatic disease model, however, the size of the object under investigation (the mouse/rat) can be very large relative to the size of the structures of interest (the tumors). When attempting to capture both global and local scales in an image, this translates into a large field-of-view at high image resolution, resulting in images of tens of millions of voxels. In such cases, solving the SRR problem becomes very expensive, in terms of both computation time and memory requirements. Exploring large data sets in this way calls for conceptual new-thinking.

In this study, we propose a novel approach to overcome the computational issues of whole-body SRR without sacrificing reconstruction quality. By integrating state-of-the-art methods from the areas of articulated atlas-based segmentation of whole-body small animal data [5]–[9], planar reformation [10], and SRR in MRI [3], [11], we arrive at a novel localized approach to SRR that enables interactive global-to-local exploration of *e.g.* whole-body mouse MRI data while being computationally efficient. The idea is similar to that of well-known web-based geographical maps, where it is possible, from a global overview image, to zoom in on a detail of interest. Guided by user interaction or by registration to images of higher sensitivity, such as BLI, local volumes-of-interest (VOIs) can be identified in the low-resolution MR image and enhanced by SRR to show a higher level of detail.

Thus, the goals of this work are two-fold:

1. To provide an integrated, interactive platform for local super-resolution reconstruction of MRI whole-body mouse data.
2. To demonstrate in a proof-of-concept study that local SRR is a feasible method for improving visualization and localization of metastases in whole-body small animal imaging studies, where by feasibility we refer to the following two aspects:
  - *Image quality:* Does the local SRR method improve the visualization of small anatomical details over conventional imaging methods, under the condition that the number of low-resolution images used for the SRR is constrained by a total acquisition time compatible with *in vivo* experiments?
  - *Computational feasibility:* Can the local SRR computations be handled on a desktop machine in a close-to-real-time time frame?

In the following sections, we first introduce our approach to local SRR in MRI. We briefly describe its components (for details we refer to previously published work in which each of the components has been thoroughly validated) and present a phantom experiment that quantifies the ability of SRR to detect micro-structures. We validate our approach in two case studies with bone and kidney breast cancer metastases visualization and finally discuss the presented results.

## Materials and Methods

### Experimental mouse model and imaging

To test the SRR approach, BLI, CT, and MRI were acquired in a mouse model of metastasizing breast cancer. One female, *Balb/c nu/nu* mouse of 19.5 g was used. At 7–8 weeks of age, the mouse was injected with 4T1-luc2 [12], [13] breast cancer cells (100 µl, 150,000 cells) into the left heart ventricle under 2% Isoflurane anesthesia.

After 2–3 weeks, BLI and CT scans were made *in vivo*. The anesthesia applied was Ketamine∶RomPun∶PBS (1∶1∶1), approximately 60 µl/20 g. This was followed by an *ex vivo* MRI scan. The mouse was euthanized to allow flexibility in the MRI experiments and test different acquisition parameters.

**Figure 1:**
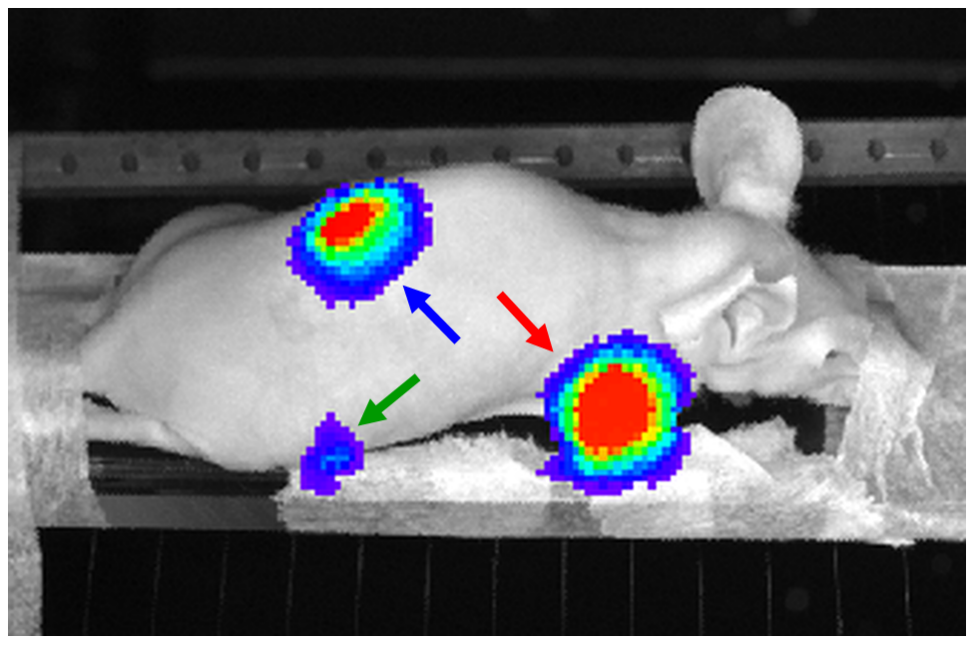
A BLI photographic image of the mouse acquired to validate the proposed approach. The arrows indicate the different tumor locations: humerus (red), femur (green), kidney (blue).

The mouse, in prone position, was taped to an in-house made PMMA holder that was used in all three scanners. BLI data was acquired using an IVIS 3D BLI Imaging system (Caliper Life Sciences, Alameda, CA). BLI images were taken from 8 positions around the animal with an exposure time of 10 s per image, allowing for 3D data reconstruction. One of the eight BLI images is presented in Figure 1.

CT data was acquired on a SkyScan 1076 *in vivo* microCT scanner (Aartselaar, Belgium) at a resolution of 35 µm. The acquisition was performed with a step size of 1.4° over a trajectory of 360° (Voltage = 49 kV, Current = 200 uA, Exposure time = 100 ms, Filter: AL. 0.5 mm, Frame averaging = 1).

Several strategies can be adopted when acquiring MR data for an SRR experiment. By acquiring the low-resolution slice stacks with rotational increments around either the frequency or the phase encoding direction, as introduced in [14], a more effective sampling of *k*-space is achieved than by shifting the low-resolution images by sub-pixel distances along the slice selection direction [3]. In this way, a whole-body scan of the *post mortem* mouse was acquired on a 7T Bruker Pharmascan system using a fast spin echo (FSE) sequence (TR = 5300 ms, TE = 53.2 ms, with N_avg_ = 4). The 2D slice stack consisted of 40 slices (0.5 mm thick), with a FOV of 70×45×20 mm^3^, and a resulting resolution of 0.125×0.125×0.5 mm^3^. The scan time per stack was 13 min. The slice stack was acquired at 24 angles with uniform increments of 180°/24 = 7.5° around the phase encoding direction. First results obtained using 24 low-resolution images were published in [9]. In this study, we performed SRR on subsets of two and four low-resolution images. In the subset of two images, the angular increment between them was 180°/2 = 90° and in the subset of four images it was 180°/4 = 45°.

Animal experiments were approved by the local committee for animal health, ethics and research of Leiden University Medical Center.

### Integrated, Interactive Local SRR Reconstruction

**Figure 2:**
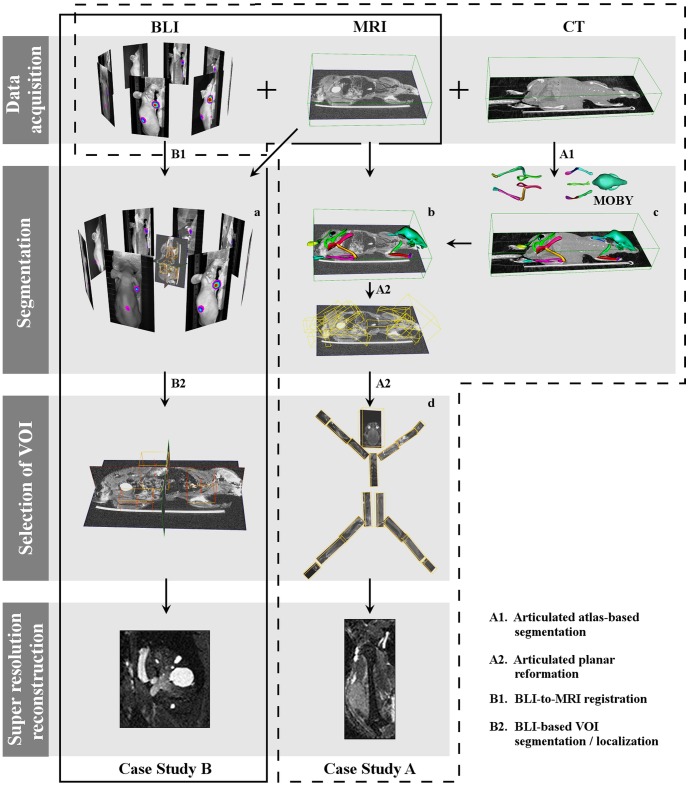
Overview of the integrated, interactive local SRR of MRI mouse data applied to two case studies. **Case Study A:** After rigidly registering CT to MRI, articulated atlas-based segmentation is performed (A1). Subsequently, articulated planar reformation is applied to the segmented MRI, and the data is visualized in the standardized atlas space (A2). The user can now interactively select any bone of interest guided by the BLI images for SRR reconstruction. A high-resolution SRR image of the humerus with a tumor is presented. **Case Study B:** BLI+MRI mouse data are first co-registered (B1) to define the VOIs (B2) using the BLI. A VOI is interactively selected for performing SRR. A high-resolution SRR image of the kidney with metastases is presented.

The local SRR method integrates a series of processing and analysis steps, which depend on the available complementary data (CT, BLI, *etc.*) and vary in their level of user interaction. The overview of the presented method can be seen in the flowchart depicted in Figure 2. First, within a set of low-resolution MRI images, potential VOIs are identified. In our approach, this step is either based on user input or it is automated, as described below. Its output is one or multiple VOIs containing potentially relevant structures. Each of these VOIs can now be selected for subsequent local SRR.

The methods for segmentation and selection of VOIs are highly specific to the biological problem and to the available complementary data. In the following, we present two situations typical in small animal tumor imaging, in which BLI+CT (Case Study A) and BLI only (Case Study B) are used as complementary modalities to MRI (see Figure 2). Each situation presents a different level of automation and requires a different degree of user interaction. The way the relevant information is extracted differs with the choice of the imaging modalities for the study at hand. In Case Study A, the level of user intervention is minimal. The whole-body mouse is automatically segmented using an articulated atlas. Guided by the BLI, the user can then select the VOIs with tumors for further SRR reconstruction, visualize the results side-by-side with the CT data, and, in case a tumor is present near a bone on one side of the body, compare it to the contralateral side, where most likely there is no tumor. In Case Study B, user interaction is necessary to co-register the BLI to the MRI data to define the VOIs. After that, the user can select among the VOIs in which the BLI signal indicate the presence of tumors for SRR reconstruction and further high-resolution visualization and analysis.

### Case Study A: MRI+CT+BLI

This case study was set up to explore the applicability of local SRR-MRI to image bone metastases as a complementary modality to CT, BLI and conventional MRI. In this section, we describe our approach to super-resolution bone MRI.

#### Articulated atlas-based bone segmentation of CT and MRI mouse data

First, rigid registration of the CT scan to one of the low-resolution MR images was performed [7], [15]. Rigid registration was sufficient in this case because the mouse was fixated in the same animal bed during all imaging procedures and during transport between scanners. The bones were segmented in the CT image using the articulated MOBY mouse atlas [6], [16] (Figure 2.c). The fully automated segmentation approach presented in [5] was used for this purpose. To deal with the large articulations between bones and/or bone groups, the registration of the atlas to the CT data used a hierarchical model tree. To begin with, a coarse alignment of the MOBY atlas to the CT skeleton was performed. This was followed by the stepwise alignment of the individual atlas bones to the CT data, using the ICP algorithm [17]: we started with the skull, after which each bone was accurately registered to the corresponding bone in the data. Given the CT-to-MR registration parameters, the transform obtained in the segmentation of the whole-body CT data was propagated to the MR. Figures 2.(b, c) show the atlas fitted to the CT and MRI datasets, respectively; see [5] for more details.

#### Articulated planar reformation of MRI data

Using the obtained transformations between each bone in the atlas and the low-resolution MR image, articulated planar reformation [10] can be applied to map the labeled data into a standardized atlas space. This method automatically creates for each bone a VOI, which is based on a principal component analysis of the bone shape. By constructing the VOIs in this manner, the final reformatted images are aligned with the principal axes of the bones [10].

#### (Interactive) selection of VOIs

Upon segmentation and reformation, the user is presented with a global view of the segmented bones; see Figure 2.d. From this view, the user can choose bones of interest and perform local SRR on them. Alternatively, the BLI-signal can be used to automatically identify VOIs with tumors for SRR reconstruction.

### Case Study B: MRI+BLI

This case study was set up to explore the value of SRR-MRI as a complementary modality to BLI and conventional MRI when CT data is not available for establishing anatomical correspondence. In practice, this is usually the case for soft tissue tumors, where bone metastases and bone resorption are not expected. In this section, we describe our approach to local super-resolution MRI of kidney tumors.

#### BLI-to-MRI mouse data registration

After acquisition, the BLI images are registered to one of the low-resolution MR images using a landmark-based approach [7], [15]. A minimum of three landmarks is selected. The location of each landmark is indicated in one of the low-resolution MR images and in two separate BLI images at different angulations. Using the known angle between the two BLI images, back-projection is applied to find the corresponding point in the three-dimensional space. This point is then paired with the point in the MR image, and registration is performed. Typical landmarks include the snout and limbs as they are most easily identified in both modalities.

#### BLI-based VOI localization and extraction

VOIs can be identified by simple thresholding on the raw BLI signal. Once the coordinates of the VOIs in world space are known, the BLI-to-MRI registration transform is used to map the VOIs onto the chosen low-resolution MR image. The MRI VOIs are then propagated to the remaining low-resolution MRI images using the transform parameters of these acquisitions. Finally, corresponding VOIs are extracted from all LR images and used for SRR. This step is performed automatically.

### Super-resolution reconstruction

When a VOI has been selected and propagated to all low-resolution MRI images, local SRR can be performed on that volume.

SRR is the process of producing a single high-resolution image from a sequence of low-resolution images, where each low-resolution image transforms and samples the high-resolution scene in a distinct fashion. It is an inverse problem in which the acquisition process is modeled as a linear operator on the high-resolution image. When the high-resolution image is vectorized and put into a large vector **x**, the acquisition of the low-resolution image *k* can be modeled as **y** *_k_* = **A** *_k_* **x+n** *_k_*, where **n** is Gaussian noise [18]. The linear operator **A** *_k_* models the transform due to the rotation of the field-of-view of the *k* ^th^ image as well as the point spread function of the acquisition.

The objective in SRR is to find an **x** that simultaneously minimizes the difference between **y** *_k_* and **A** *_k_* **x** for all *k* [3]. In general, a direct solution of this objective is not feasible since it involves many operations with all 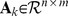, where *n* and *m* are the number of voxels in the reconstruction (**x**) and in a low-resolution image (**y** *_k_*), respectively. Instead, the reconstruction is obtained by iterative methods such as the conjugate gradient method. In this study, we apply the method described in [11], which uses the conjugate gradient method to solve the Tikhonov-regularized least-squares problem and implements **A** and **A** *^T^* by an affine transformation scheme that minimizes aliasing and spectral distortion. The SRR method is extended with a bias-field correction step removing inhomogeneity differences between the images caused by variations in coil sensitivity.

### Phantom validation study

To quantitatively evaluate the performance of SRR with respect to detection of micro tumors, we designed a phantom experiment in which micro tumors were simulated by fluorescent micro beads, and imaged using a very similar acquisition protocol to the one described in “Experimental mouse model and imaging” section. In the following we describe the phantom, the imaging setup, and our quantification method in full detail.

**Figure 3:**
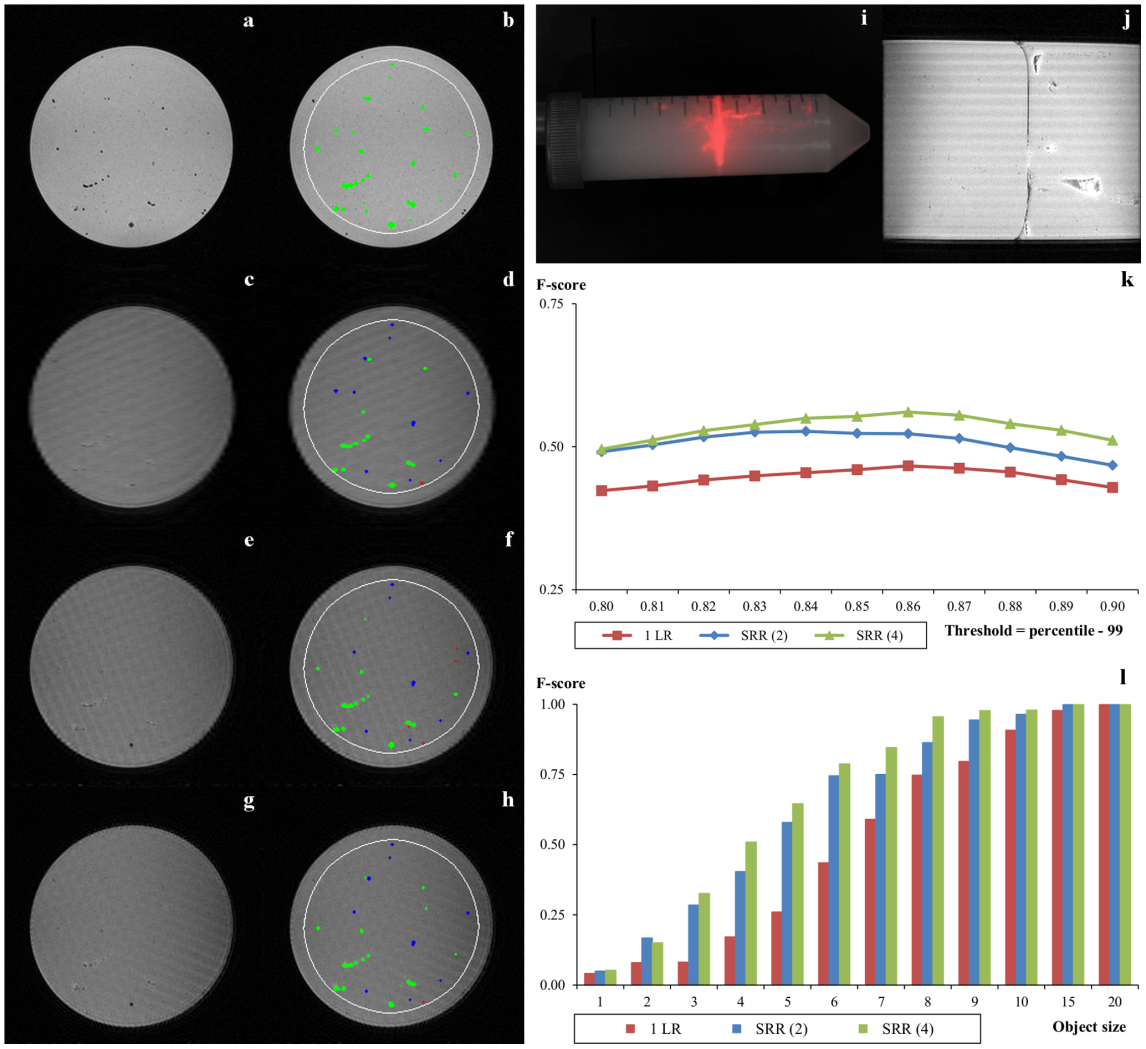
Phantom experiment. (a, c, e, g) One transversal slice from the 3D GE image (gold standard), single low-resolution (1 LR), SRR (2), and SRR (4) reconstructions respectively. (b, d, f, h) The same slice with the boundary of the region used for quantification (white) and detected objects (green, red, and blue; all regions occupied by segmented objects have been dilated with one pixel for better visualization). Green color indicates true positive detections based on the high-quality GE scan, red—false positives, and blue—false negatives (note that several visually missing objects were detected on other slices, hence they are not highlighted as false negatives on the shown slice). (i) FLI image of the phantom. (j) One coronal slice from the 1 LR image. (k) Detection performance in terms of the F-score of different reconstruction methods as functions of the threshold on the bottom-hat image. (l) F-score as function of object size for optimal threshold on each of the reconstructed volumes.

Different-sized clusters of fluorescent beads were immersed in agar and used to simulate the micro metastases. Fluorescence imaging (FLI) was used instead of BLI (Figure 3.i), and a 3D gradient echo (GE) sequence was used as the gold standard (Figure 3.a). The use of this sequence was possible due to the low proton density of the fluorescent beads. The SRR results were quantitatively compared to that of the interpolated single low-resolution image and the 3D GE.

#### Phantom preparation

Red fluorescent polyethylene microspheres with a diameter of 150–180 nm (UVPMS-BR-0.995, Cospheric LCC, Santa Barbara, CA, USA) were left overnight in 2 ml 1‰ Tween (Sigma-Aldrich, St. Louis, MO, USA) in a 2 ml reaction vial (Eppendorf, Hamburg, Germany) on a roller bank in a concentration of 250 mg particles per ml in order to get single particles in suspension.

Approximately 25 ml 2% agar (Sigma-Aldrich) was poured into a 50 ml conic centrifuge tube (Sigma-Aldrich) and left at room temperature to cool down. Small volumes (10–20 µl) of microsphere suspension were carefully pipetted underneath the agar surface after the agar polymerization started, but before the agar gel was completely set. The rest of the tube was filled with 2% agar after the initial 25 ml agar polymerized (Figure 3.i).

#### Data acquisition

FLI of the phantom was performed using the IVIS Spectrum (PerkinElmer, Waltham, MA, USA) using the λ_ex_ = 430 nm and λ_em_ = 600 nm filters (Figure 3.i).

Four low-resolution MRI views of the phantom, rotated 45° with respect to each other, were acquired with a protocol very similar to the one described in the “Experimental mouse model and imaging” section: 64 slices, in plane resolution 0.1305×0.1305 mm^2^, slice thickness 0.52 mm and a FOV 33×33×33 mm^3^. In addition, high-quality GE image of the phantom was acquired with the same resolution as the low-resolution images and used as the gold standard.

#### Object detection

The SRR images using 1, 2, and 4 low-resolution volumes (labeled 1 LR, SRR (2), and SRR (4) respectively) were calculated as explained in the “Super-resolution reconstruction” section. F-score of the detected objects (separate individual beads or bead clusters), defined as F-score = 2×precision×recall/(precision+recall) was used as quantitative measure of the quality of the three reconstructed volumes. The objects were detected by using a bottom-hat filter with a structuring element in the form of a 5×5 square, followed by thresholding and extraction of connected components [19]. Initially, multiple thresholds were defined as an intensity percentile of the corresponding bottom-hat map, and tested on the LR image, the SRR images, and the gold standard GE image; see Figure 3.k. For each data set, the threshold maximizing the F-score was chosen as the optimal one. In this analysis, objects located close to the boudary of the phantom were disregarded to exclude possibility of incorrect detection in this region due to reconstruction artifacts.

### Hardware and Software platforms

The experiments described in the sections above were performed on a 2.80 GHz Intel Xeon with 12 GB of RAM Windows Workstation. The described registration, segmentation, and SRR algorithms were implemented in MATLAB R2009b.

## Results

### Case Study A: MRI+CT+BLI (bone tumors)

Local SRR images of the right femur and humerus with metastases were reconstructed at different levels of quality using 2 and 4 low-resolution images and compared with a single low-resolution (1 LR) MRI image, BLI, and CT. In addition, reconstruction times of individual bones were compared with that of the entire mouse.

**Figure 4:**
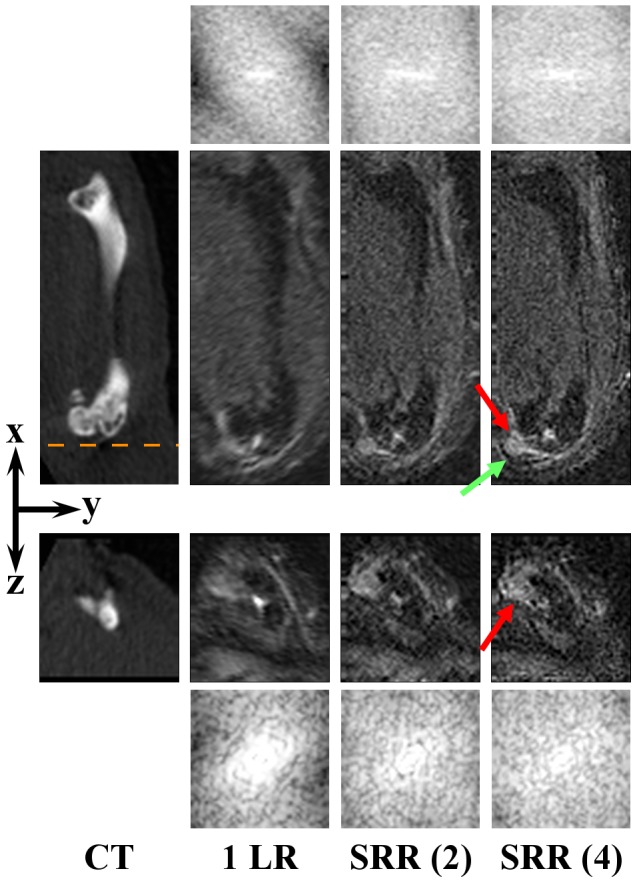
Right femur. From left to right: a CT scan, a single low-resolution image (1 LR), and SRR reconstructions, each based on a different number of low-resolution images. Two orthogonal slices of the same VOI are shown to illustrate the effect of the SRR in a 3D volume. The orange dashed line approximately indicates where the *yz*-slice (3^rd^ row) intersects the *xy*-slice (2^nd^ row). The red arrows points to the (micro) tumor in the knee. The green arrow points to a location outside the tumor, at which recovery of the fine details is obvious: the margins of the tumor are clearly delineated. The CT and all the MR images are shown in the coordinate system associated with the principal axes of the bone, and the low-resolution volume is resampled to isotropic resolution beforehand. Image contrast on the MRI images was increased for visualization purposes. For all MR images the corresponding frequency spectra up to the Nyquist frequency of the reconstruction volume are shown, above and beneath the *xy*-slices and the *yz*-slices, respectively, demonstrating enhanced high-frequency content.

On BLI (Figure 1), three distinct signal areas were observed, the smallest one at the position of the right femur (green arrow). The user therefore manually selected the right femur for SRR of the MRI data, using 2 or 4 low-resolution images for the reconstruction: SRR (2) and SRR (4) respectively; see Figure 4. The arrows in the BLI and the SRR (2) and SRR (4) images point to a tumor adjacent to the medial chondyle. This tumor is neither visible in the CT image, nor in the low-resolution image. When using 2 low-resolution images for SRR, the image quality increases, and the tumor becomes discernible. Using 4 low-resolution images further improves the visibility of the tumor and its margins.

**Figure 5:**
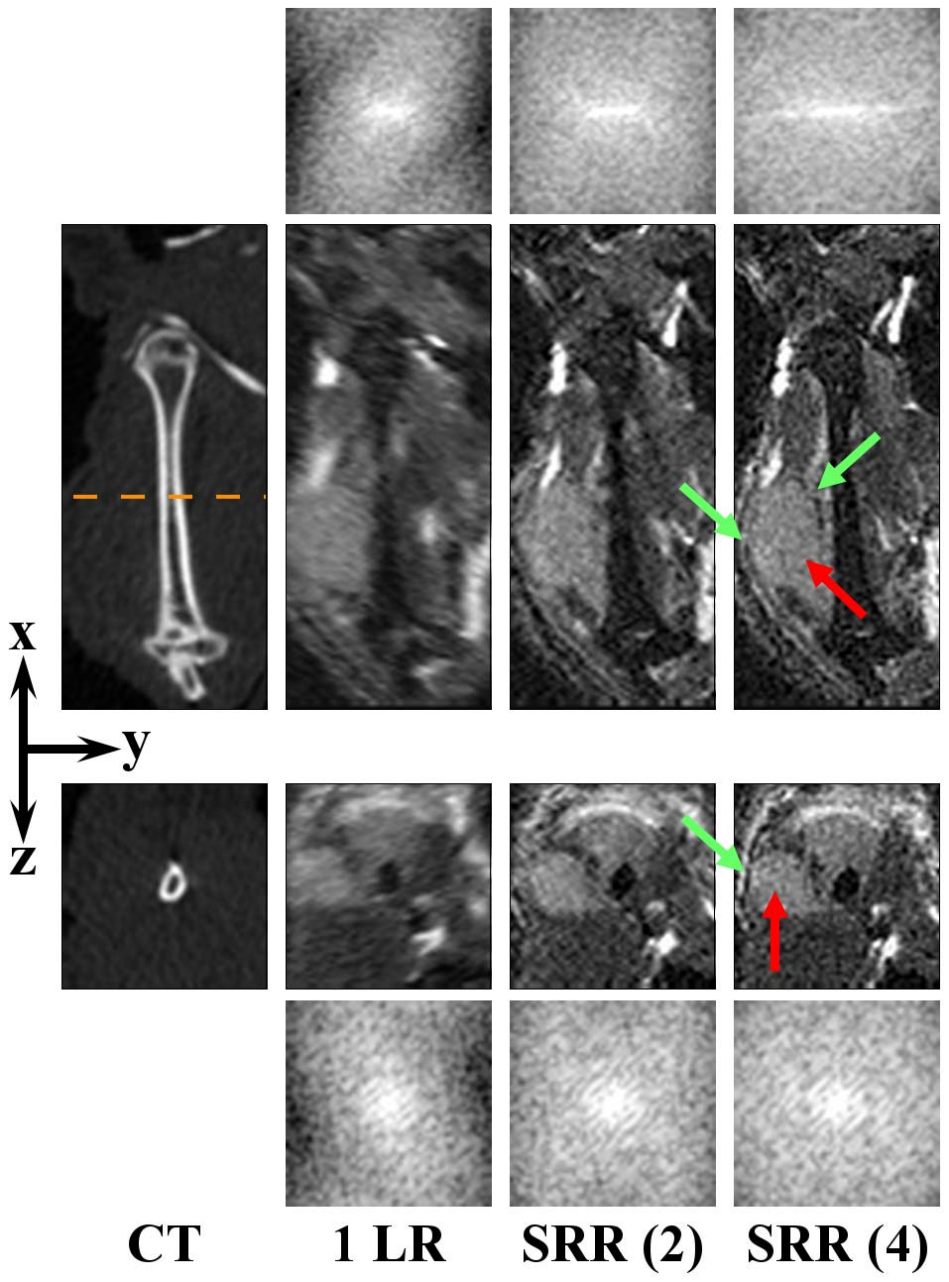
Right humerus. From left to right: a CT scan, a single low-resolution image (1 LR), and SRR reconstructions, each based on a different number of low-resolution images. Two orthogonal slices of the same VOI are shown to illustrate the effect of the SRR in a 3D volume. The orange dashed line approximately indicates where the *yz*-slice (3^rd^ row) intersects the *xy*-slice (2^nd^ row). The red arrows point to the tumor. The green arrows point to some of the locations where recovery of the fine details is the most noticeable. The CT and all the MR images are shown in the coordinate system associated with the principal axes of the bone, and the low-resolution volume is resampled to isotropic resolution beforehand. Image contrast on the MRI images was increased for visualization purposes. For all MR images the corresponding frequency spectra up to the Nyquist frequency of the reconstruction volume are shown, above and beneath the *xy*-slices and the *yz*-slices, respectively, demonstrating enhanced high-frequency content.

BLI also showed a high intensity area at the location of the right humerus (Figure 1, red arrow). The tumor is not visible on CT (Figure 5). The single low-resolution image does show the tumor, but, due to the relatively thick slices, the tumor margins are blurred, particularly in the transverse plane. As before, the image quality improves when using more low-resolution images, showing a clear delineation of the tumor, with SRR (4) being sharper and less noisy than SRR (2).

To assert that SRR produced an actual resolution enhancement, frequency spectra were produced by applying a windowed Fourier transform to the shown MRI slices. The resolution enhancing effect in Figures 4 and 5 is clear: when more images are used for reconstruction, the spectrum of the SRR image contains more high-frequency content than the low-resolution images.

Table 1 shows how the SRR reconstruction times scale approximately linearly with the size of the low-resolution dataset. Since a single low-resolution image of the entire mouse contains approximately 20 million voxels, and a typical VOI contains around 250,000 voxels, we accelerate the reconstruction by approximately a factor 80. From the table, it also follows that the SRR times scale approximately linearly with the number of low-resolution images used. While the entire mouse takes more than 40 minutes to reconstruct using 4 low-resolution images, a VOI can be reconstructed within 1–2 minutes.

**Table 1:** SRR times in seconds for each reconstructed right bone and the whole-body of the mouse, using 2 and 4 low-resolution images.

	2 low-resolution	4 low-resolution
Femur	56	98
Tibia-Fibula	38	75
Pelvis	79	151
Sternum	31	63
Humerus	48	83
Ulna-Radius	41	78
Whole-Body	1282	2479

The segmentation and selection of VOIs steps described above each take less than a minute to perform.

### Case Study B: MRI+BLI (kidney tumors)

**Figure 6:**
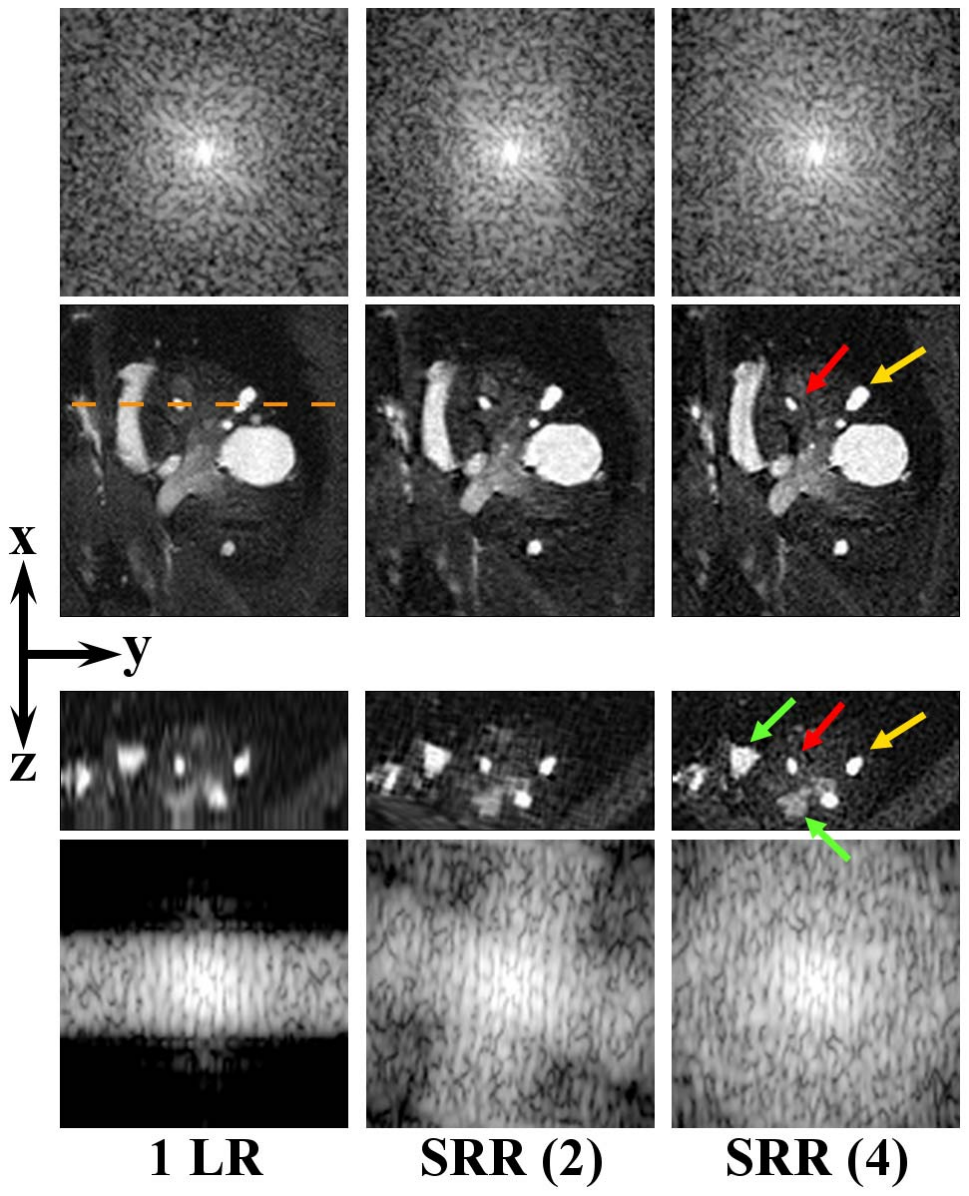
Right kidney. From left to right: a single low-resolution image (1 LR), and SRR reconstructions, each based on a different number of low-resolution images. The red and yellow arrows point to two different tumors. Two orthogonal slices of the same VOI are shown to illustrate the effect of the SRR in a 3D volume. The green arrows point to other locations where the improvement in image quality is particularly noticeable. The orange dashed line approximately indicates where the *yz*-slice (3^rd^ row) intersects the *xy*-slice (2^nd^ row). In all the MR images, the *xy*-view is the in-plane direction of the scans. Note that the metastatic lesion seen in the BLI image (Figure 1, blue arrow) actually consists of numerous lesions as shown on MRI scans. For the low-resolution image, the selected views are resampled to isotropic resolution and the image contrast on the MRI images was increased for visualization purposes. For all MR images the corresponding frequency spectra up to the Nyquist frequency of the reconstruction volume are shown, above and beneath the *xy*-slices and the *yz*-slices, respectively, demonstrating enhanced high-frequency content.

BLI showed a single high signal intensity around the area of the right kidney (Figure 1, blue arrow). Local SRR images of this area were reconstructed at different levels of quality and compared with a single low-resolution MRI image and BLI. Figure 6 shows orthogonal slices of the kidney for the different image types (a single low-resolution image, and SRR on 2 and 4 low-resolution images). On the BLI in Figure 1, the spatial resolution is too low to determine whether multiple tumors are present, but on MRI one large possibly cancerous lesion and several small suspected lesions can be detected. Most of these are readily detectable on the low-resolution image. However, the lesions appear blurred and cannot be clearly delineated. In such images, the smallest lesions are lost due to partial volume effects, but will be recovered in the SRR (2) or SRR (4) images. The high 3D resolution of the SRR scans also shows that most of these suspected lesions are located in the renal cortex and medulla, whereas the renal pelvis is relatively clean. Again, we asserted the resolution enhancement due to SRR by producing frequency spectra of the shown images.

### Phantom quantification

A comparison of Figure 3.i and Figure 3.j clearly shows that it is impossible to distinguish the different clusters of beads (simulated micro tumors) using FLI due to its limited spatial resolution. It is, however, possible to distinguish the clusters using MRI.

From the plots shown in Figure 3.k, it follows that detection on both SRR volumes is, in general, much better in comparison with the interpolated 1 LR volume. SRR (4) exhibits consistently better object detection in comparison with SRR (2) and 1 LR.

Figure 3.l illustrates detection scores on each reconstructed volume as a function of the object size. Performing this analysis with increasing object size threshold shows an increasing trend for the detection score on all volumes, with perfect detection rates for all three volumes on objects larger than 20 voxels. This analysis shows that SRR greatly improves detection of small objects. Moreover, using more LR images for reconstruction results in higher detection rates, where the quality improvement is attributed to better performance on small objects.

## Discussion

In this paper we have presented a proof-of-concept study as a first step towards real-time, interactive local SRR-MRI. While the approach can be applied in many biomedical imaging settings, where both global and local scales are relevant, we have chosen to explore our method in the context of bone and kidney metastases in mice. In the following, we discuss the results of the study and deliberate on potential challenges as well as advantages of the method.

### Relevance to tumor research and other biological applications

Conventionally, bone resorption and metastases in soft tissues (such as kidney, lung, and liver) are visualized using BLI+CT and BLI+MRI, respectively. In this study, we have explored the value of adding SRR-MRI to improve soft tissue tumor detection. We have shown in two case studies how an integrated approach, combining state-of-the-art technologies from the area of image processing with the use of multiple imaging modalities, can be used to detect and study bone and soft tissue metastases with much greater sensitivity than by the conventional methods.

In Case Study A, we saw how BLI is a sensitive method to visualize luciferase-positive tumors in a living animal. The BLI signal intensity is proportional to the size of a tumor mass, and BLI can thus be used to give a rough estimate of both size and location of the lesion. In the case of bone metastases, the location and subsequent bone pathology are usually determined using CT [20]. However, in Case Study A there was no visible bone pathology in the CT scan. When local SRR-MRI guided by the BLI signal was performed, these images provided the location, size, and shape of tumors in the limbs of the animal and confirmed that these metastases were, indeed, soft tissue tumors located outside of the bone. In the low-resolution MRI images of the femur, the tumor could not have been identified without the guidance of the BLI images. The SRR-MRI, on the other hand, clearly showed a nodular structure that could be identified as a tumor (Figure 4). In the humerus images, which contain a large tumor outside of the bone, it can be appreciated how the delineation of the tumor boundary becomes much sharper in the SRR-MRI than in the single low-resolution MR image (see Figure 5; note that the improvement in image quality is especially noticeable when using a high zooming factor). The method thus has the potential to support detailed quantitative studies of *e.g.* metastases development and assessment of treatment response.

In Case Study B of kidney metastases, CT was not used, as this modality gives insufficient soft tissue contrast without the use of contrast agents. BLI indicated the presence of a cancerous lesion in or around the kidney (Figure 1, blue arrow). MRI revealed numerous independent metastases in the kidney (Figure 6), which is not possible with BLI alone due to its limited spatial resolution. Moreover, SRR-MRI allows the researcher to not only distinguish, but also to clearly delineate different tumors in close proximity. This cannot be achieved with conventional MRI, as illustrated in Figure 6 and evaluated quantitatively on phantom data. SRR-MRI can thus provide added value in studies where the number of metastases is an important parameter and where experimental treatment is used to intervene with the metastatic process. For instance, a researcher can differentiate between renal, adrenal, and peri-renal cancerous lesions with SRR-MRI but not with BLI.

BLI remains the preferred standard measurement for active tumor size as the signal originates only from living cells and not from a necrotic core or cells killed by a certain treatment. Light, however, only has a limited penetration in bone, which, in turn, can mask the BLI signal coming from small tumors that grow inside it, making these tumors appear smaller than they actually are. Having an MRI dataset, in which the tumor can be identified and clearly measured, helps overcoming these limitations.

An additional point to be made is the possibility to use BLI with SRR-MRI as an alternative for the CT anatomical reference, particularly in longitudinal studies where the repeated exposure to radiation in a CT scan may become a confounding factor or cause adverse effects [21].

Apart from oncology, the presented work flow may be of value in many research areas that requires whole-body examination for local ((sub-) slice-thickness sized) effects. Examples are the homing of labeled stem cells after systemic injection, or imaging of systemic inflammatory diseases.

### Post mortem to in vivo SRR-MRI

In this study, we have applied our approach to *post mortem* MRI data. However, we have well-founded reasons to assume that our results translate to *in vivo* MRI imaging, because the acquisition protocol used in these experiments is compatible with *in vivo* mouse imaging. The main difference that can be expected between *ex vivo* and *in vivo* acquisitions is the presence of motion. Motion-induced artifacts are reduced by fast low-resolution acquisitions and accurate subsequent registrations. While accurate non-rigid registration of soft tissue structures, such as liver and kidney, may be possible, SRR is expected to be most successful for relatively rigid structures, such as the brain, bone, and tissue surrounding bone: cases in which rigid registration will yield accurate alignment of the low-resolution images. In [3], we showed SRR reconstructions of an *in vivo* mouse brain, and several studies have validated the assumption of accurate motion estimation in applications of SRR in fetal brain MRI [22], [23].

### Interactive local SRR

One of the contributions of this work has been the development of an approach that integrates recent progress in the areas of articulated atlas-based segmentation of whole-body small animal data, planar reformation, and SRR in MRI into a novel localized approach to SRR that enables global-to-local exploration of *e.g.* whole-body mouse MRI data. Together with the preliminary results first published in [9], we have provided a global solution to three possible scenarios that takes into account the availability of complementary data: (*i*) only MRI is available [9], (*ii*) MRI+BLI is available, (*iii*) MRI+CT+BLI is available. From first to last scenario, the proposed approach decreases in the required level of user interaction to segment the data into possible VOIs. Depending on the biological problem, the more complementary data available, the higher the level of automation of the approach and the more data can be provided for the user to explore, *i.e.:* in the approach of Case Study B (MRI+BLI), the user can choose only among VOIs in which BLI signal is present for a subsequent SRR reconstruction. Alternatively, (if CT is available) the user can select any bone for the SRR reconstruction and thus compare left with right, a bone with a tumor with the same bone without a tumor on the contralateral side, *etc.* Naturally, the more complementary data available in a study, the more information one can extract. Thus, while in (*i*) only MRI information is available, in (*iii*) one can fully integrate the information provided by the BLI (which quickly locates tumor growth and indicates tumor burden) together with the anatomical information provided by the CT (used to study tumor-induced changes in the bone—bone resorption) and the soft tissue information provided by MRI (which can provide the information about the size and the number of metastases).

### Image quality vs imaging time

A major constraint when applying SRR in small animal MRI is the limited acquisition time that *in vivo* experiments allow. Each of the low-resolution images takes a certain amount of time to acquire and acquisition of multiple such images may quickly exceed the time during which a mouse can be kept sedated. It was shown in [3] that relatively few images are necessary to achieve significant improvements in the image quality. In this study, we have limited the number of low-resolution images to four, with a total acquisition time of 52 minutes – a realistic acquisition time for *in vivo* experiments. If the experimental setting allows it, the number of low-resolution images used can be extended at the expense of additional acquisition time. This will have some positive effect on the resolution. For an optimal coverage of *k*-space, the number of low-resolution images should be ⌈π/2×F⌉, where F is the anisotropy factor, *i.e.*, the slice thickness relative to the in-plane resolution. In our case, that would mean using 7 low-resolution images. Using more than this number of low-resolution images will not have a significant impact on the resolution, but will increase the SNR slightly (for an in-depth study of these trade-offs, we refer the reader to [3], [4]).

The major advantage of SRR in small-animal MRI is that it enables obtaining isotropic images in scenarios where T2-weighted image contrast is desired, requiring long repetition times and therefore long scan times, particularly for a 3D acquisition. By combining a small number of relatively fast thick-slice acquisitions with SRR, an isotropic resolution close to the original in-plane resolution is obtained. For comparison, direct acquisition of a 3D fast spin echo image with the same resolution and acquisition parameters would take about 28 hours and thus is infeasible. Alternatively, accelerating the 3D acquisition to similar scan times by reducing TR and increasing the echo train length leads to severe blurring at high field strength due to the relatively fast T2 decay. The proposed SRR solution would also be suitable on advanced acquisition schemes for high-field abdominal imaging. For instance, using SRR in combination with a self-navigated acquisition scheme like PROPELLER should provide high-resolution 3D T2-weighted contrast at high fields while reducing motion artifacts [24], [25].

### Reconstruction times

For large datasets, the SRR method is limited by the memory available on the computer. For the conjugate gradient solver, up to 5 data structures, each the size of the final reconstructed image, and 2 additional data structures, each the size the total low-resolution data, must be kept in memory simultaneously. For large 3D data sets, this soon becomes very difficult to achieve, even on a high-performance desktop computer. The interactive approach to locally reconstruct VOIs presented here, allows overcoming the time and memory limitations of the SRR technique. However, as shown in Table 1, the mean time for the best quality SRR result, *i.e.*, using 4 low-resolution images, is still in the order of minutes—91.3 s. The mean time for SRR using 2 low-resolution images is 48.8 s. These results are still far from the real-time target for this approach. Since the results presented here were acquired on a MATLAB implemented prototype, the computation times will decrease by re-implementing the algorithm in a C/C++ and GPU combination.

## Conclusions

By combining a number of state-of-the-art image processing techniques, we have enabled a global-to-local exploration of whole-body mouse MRI. We have shown that the SRR-MRI is a valuable complementary modality in studies of tumor metastases. Using only a few low-resolution images, and a total acquisition time compatible with *in vivo* experiments, we have reconstructed SRR MR images from which detailed information about soft tissue metastases, not available in conventional imaging modalities, can be inferred. This cannot be obtained from direct MR acquisition within a feasible acquisition time.
